# Account of Diseases in an Eastern District of London, from the 20th of November to the 20th of December, 1807

**Published:** 1808-01

**Authors:** 


### *Account of Diseases in an Eastern District of London, from the 20th of November to the 20th of December,* 1807.

| | | | |
|---|---|---|---|
| ACUTE DISEASES. | | Cephalalgia - - | 7 |
| Pneumonia - - | 3 | Vertigo - - - | 3 |
| Peripneumonia Notha - | 7 | Dysuria - - - | 4 |
| Peritonitis - - | 2 | Dysmenorrhœa - | 3 |
| Rheumatismus Acutus | 2 | Rheumatismus Acutus | 17 |
| CHRONIC DISEASES. | | PUERPERAL DISEASES. | |
| Tussis - - - | 15 | Ephemera - - | 4 |
| Tussis cum Dyspnœa - | 18 | Hæmorrhois - - | 2 |
| Dyspnœa - - | 10 | Dysuria - - | 1 |
| Catarrhus - - | 6 | Menorrhagia Lochialis | 3 |
| Phthisis Pulmonalis - | 3 | INFANTILE DISEASES. | |
| Hydrothorax - - | 1 | Diarrhœa - - | 3 |
| Pleurodyne - - | 6 | Aphthæ - - - | 2 |
| Hepatitis Chronica - | 2 | Dentitio - - | 1 |
| Icterus - - | 1 | Herpes - - - | 2 |
| Diarrhœa - - - | 3 | | |

The season has now arrived, in which we have, generally, occasion to announce a very large proportion of those diseases, which have their seat in the chest. The last few weeks, indeed, have exhibited so great a variety of seasons, that several constitutions, not particularly subject to those complaints, have been unpleasantly affected. Coughs, colds, and peripneumonies, have, however, been more frequent than any other.

Patients labouring under phthisis pulmonalis, have been much affected by the quickly changing temperature. The frequent recurrence of a very fine day has enlivened their spirits, and tempted them to try the strength of their limbs in the open air; but they have generally found the experiment succeeded by an increased irritation of the lungs, and have paid too dearly for so transient an indulgence.

INTELLIGENCE.

